# Rapid diagnostic testing for SARS‐CoV‐2: Validation and comparison of three point‐of‐care antibody tests

**DOI:** 10.1002/jmv.26913

**Published:** 2021-05-03

**Authors:** Rasmus Strand, Louise Thelaus, Nils Fernström, Torgny Sunnerhagen, Ylva Lindroth, Adam Linder, Magnus Rasmussen

**Affiliations:** ^1^ Division of Infection Medicine, Department of Clinical Sciences Lund, Faculty of Medicine Lund University Lund Sweden; ^2^ Division of Medical Microbiology, Department of Laboratory Medicine, Faculty of Medicine Lund University Lund Sweden

**Keywords:** COVID‐19, point‐of‐care test, SARS‐CoV‐2, sensitivity, specificity, validation

## Abstract

With the emergence of severe acute respiratory syndrome coronavirus 2 (SARS‐CoV‐2), a need for diagnostic tests has surfaced. Point‐of‐care (POC) antibody tests can detect immunoglobulin (Ig) G and M against SARS‐CoV‐2 in serum, plasma, or whole blood and give results within 15 min. Validation of the performance of such tests is needed if they are to be used in clinical practice. In this study, we evaluated three POC antibody tests. Convalescent serum samples from 47 reverse transcription‐polymerase chain reaction (RT‐PCR) verified patients with coronavirus disease 2019 (COVID‐19) collected at least 28 days post RT‐PCR diagnosis as well as 50 negative pre‐COVID‐19 controls were tested. The three tests (denoted the J‐, N‐, and Z‐tests) displayed the sensitivities of 87%, 96%, and 85%, respectively, for the detection of IgG. All tests had the same specificity for IgG (98%). The tests did not differ significantly for the detection of IgG. The sensitivities for IgM were lower (15%, 67%, and 70%) and the specificities were 90%, 98%, and 90%, respectively. The positive and negative predictive values were similar among the tests. Our results indicate that these POC antibody tests might be accurate enough to use in routine clinical practice.

## Highlights

The vast majority of patients with verified COVID‐19 had developed IgG antibodies that could be detected with three different point‐of‐care SARS‐CoV‐2 antibody detection tests four weeks after infection.

The tests had high sensitivity and specificity to detect anti‐SARS‐CoV‐2 IgG and but more variable sensitivity and specificity to detect IgM.

## 1 INTRODUCTION

Since its discovery in early 2020, the coronavirus disease 2019 (COVID‐19) pandemic caused by severe acute respiratory syndrome coronavirus 2 (SARS‐CoV‐2) has swept across the world in an unprecedented fashion and created a massive need for rapid and accurate diagnostic tests. The currently recommended way to diagnose *active* infection is via reverse transcription‐polymerase chain reaction (RT‐PCR) based methods. By detecting viral RNA, RT‐PCR has become the reference method to which other methods are compared. Despite this, results from some studies report varying sensitivity for RT‐PCR, especially if performed on a patient in the incubation period of the disease.^1^ An alternative way of identifying individuals who have been infected is by analyzing blood or serum for presence of SARS‐CoV‐2 specific antibodies. This can be accomplished through the use of for example point‐of‐care (POC) tests which are often of the lateral flow immunoassay type and work by detecting IgG and IgM against SARS‐CoV‐2 in serum, plasma, or whole blood. Results are often available as soon as 10 min after initiation of testing. Prior studies have shown that most patients have developed antibodies two weeks after symptom onset.^2^, ^3^ While these types of rapid diagnostic tests (RDT) may not be used to identify patients with an active infection, they can potentially be used to confirm whether or not the patient has undergone infection and developed antibodies. This can be especially valuable for finding infected individuals who did not get tested with RT‐PCR during the acute phase of infection due to exhibiting few or no symptoms. A rapid influx of POC antibody tests has hit the market. The reported sensitivity of these tests varies significantly, ranging from 39% to 100%, but the specificity is generally high, ranging from 90% to 99%.^4^, ^5^, ^6^ In this study, we have compared and validated three different POC antibody tests.

## 2 METHODS

### 2.1 Data collection

The study was conducted at the Department for Infectious Diseases, Skåne University Hospital, Lund, Sweden. Convalescent blood samples from patients with RT‐PCR verified COVID‐19 (*n* = 47) were collected at least 28 days after the RT‐PCR verified COVID‐19 diagnosis. Twenty‐three (49%) of the patients were hospitalized, of whom 11 required treatment with oxygen. The blood was allowed to coagulate for 1 h and centrifuged at 570×*g* for 10 min. Serum was frozen at −80°C until analysis. RT‐PCR for SARS‐CoV‐2 was performed on nasopharyngeal swab samples with a modified in‐house method in line with World Health Organization (WHO) guidelines as described by Corman et al.^7^ In brief, primer design and assay sequence are identical to the referred method. Our modifications constitutes changes in the thermal cycling for the E‐ and RdRP genes, utilizing 48°C for 10 min followed by 95°C for 10 min. We used an annealing temperature for the E gene of 55°C, and we used an amplification phase of 45 s for both genes. Finally, for the RdRP probe we used a concentration of 0.2 µM. The negative control group (*n* = 50) was comprised of serum samples obtained from patients 4–6 weeks after discharge from in‐hospital treatment for respiratory tract infections. Serum was collected as above between 1997 and 2007. The samples had been kept frozen at −80°C since collection.

### 2.2 Antibody testing

The three tests evaluated in this study were the SARS‐CoV‐2 immunoglobulin (Ig) G/IgM Antibody test (Colloidal Gold) from Joinstar Biomedical Technology Co. (denoted the J‐test), the COVID‐19 IgG/IgM Rapid Test Cassette (Whole Blood/Serum/Plasma) from Noviral (denoted the N‐test), and the ZetaGene COVID‐19 rapid IgM/IgG test from ZetaGene Ltd (denoted the Z‐test). The three different antibody‐tests were tested as per the instructions detailed in the user manuals provided with the tests. About 10 μl of sera was dispensed in the sample wells of the J‐ and Z‐tests whereas the N‐test required 5 μl. Two drops of diluent buffer were then added. A negative result was defined as the absence of visible G or M lines in addition to the presence of a C line (control line). A positive result was defined as the presence of a visible line for G, M, or both in addition to the C line. Results were documented within 15–20 min.

### 2.3 Statistics

Categorical data were expressed as numbers and differences between data were analyzed using the *χ* ^2^ test. Fisher's exact test was used when comparing the values of two tests to each other. The specificities and sensitivities were calculated and presented with 95% confidence intervals within parentheses. The confidence intervals were estimated with the Clopper–Pearson method. Statistical analysis was performed with, and graphs were created using, GraphPad Prism version 8.3.1. A *p* value < .05 was considered statistically significant.

### 2.4 Ethical considerations

Ethical approval was granted by the Swedish national ethics committee (2020‐01747). Blood samples from patients with verified COVID‐19 were collected after informed signed consent was obtained. Serum samples from pre‐COVID‐19 patients had been stored for research purposes as part of clinical routine. The samples were anonymized during handling in the laboratory.

## 3 RESULTS

**Figure 1:**
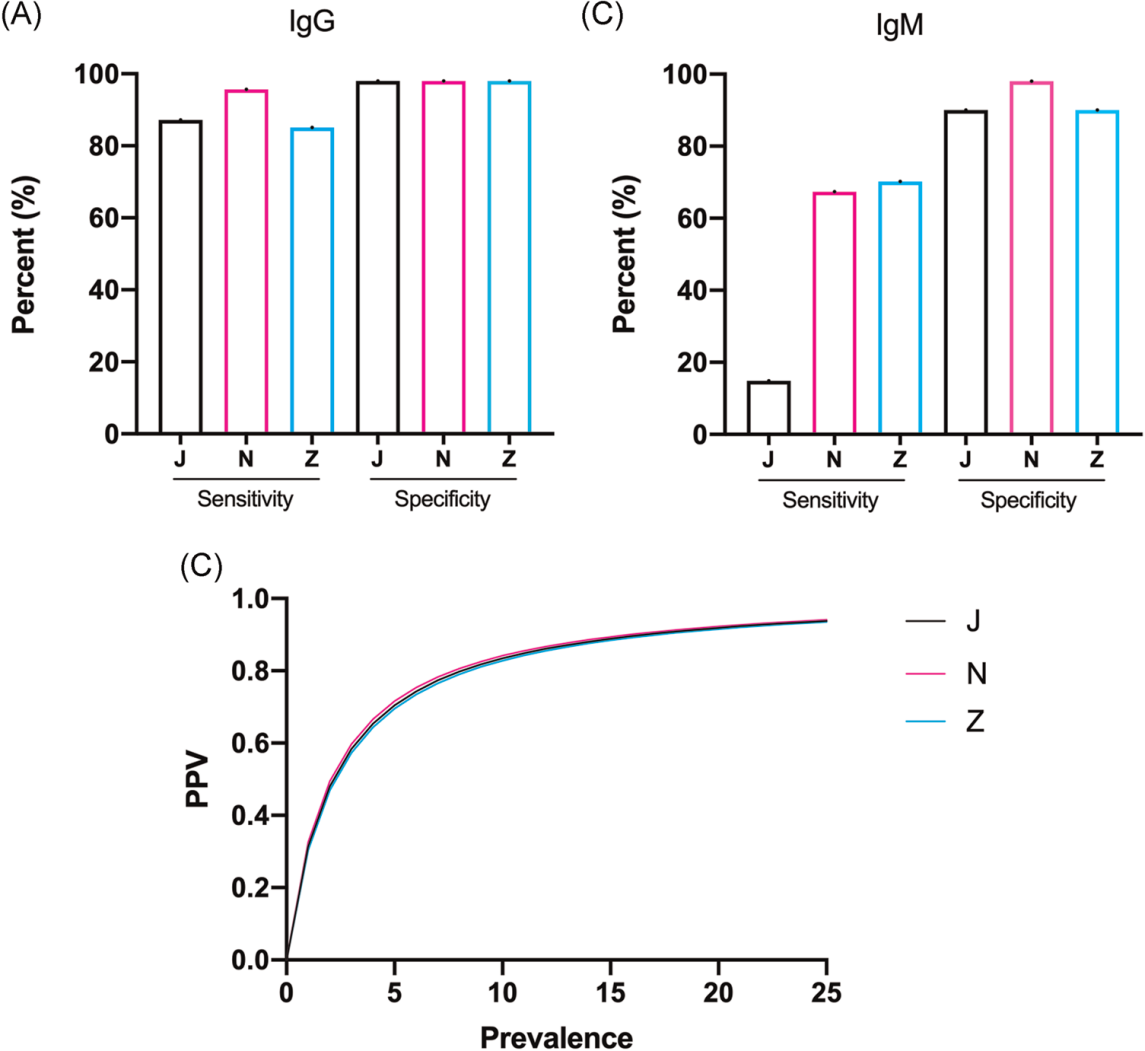
Bar and curve chart diagrams representing sensitivity and specificity for the three evaluated antibody tests. (A) Sensitivity and specificity for immunoglobulin (Ig) G antibodies found in serum samples for RT‐PCR positive patients and control patients. (B) Sensitivity and specificity for IgM antibodies found in serum samples for RT‐PCR positive patients and control patients. (C) Effect of prevalence on positive predictive value (PPV) for the detection of IgG within the range of 0%–25% for the three evaluated point‐of‐care antibody tests. J, N, and Z correspond to the SARS‐CoV‐2 immunoglobulin (Ig) G/IgM Antibody test (Colloidal Gold) from Joinstar Biomedical Technology Co., the COVID‐19 IgG/IgM Rapid Test Cassette (Whole Blood/Serum/Plasma) from Noviral and the ZetaGene COVID‐19 rapid IgM/IgG test from ZetaGene Ltd., respectively. COVID‐19, coronavirus disease 2019; RT‐PCR, reverse transcription‐polymerase chain reaction; SARS‐CoV‐2, severe acute respiratory syndrome coronavirus 2

The N‐test displayed the highest sensitivity for detecting IgG (96% [85%–99%]). The observed sensitivities for the J‐ and Z‐tests were 87% (74%–95%) and 85% (72%–94%), respectively. There was no statistically significant difference in the ability to detect IgG between the three tests (*p* = .4). All three tests displayed the same specificity (98%). Sensitivity and specificity for each test to detect IgG can be seen in Figure 1A and Table 1.

**Table 1:** Compiled values of sensitivity and specificity for IgG and IgM presented with confidence intervals

Antibody test	Sensitivity IgG	Specificity IgG	Sensitivity IgM	Specificity IgM
J‐test (%)	87 (74–95)	98 (89–100)	15 (6–28)	90 (78–97)
N‐test (%)	96 (85–99)	98 (89–100)	67 (52–80)	98 (89–100)
Z‐test (%)	85 (72–94)	98 (89–100)	70 (55–83)	90 (78–97)

As for IgM, the N‐ and Z‐tests displayed similar sensitivities: 67% (52%–80%) and 70% (55%–83%), respectively. The J‐test exhibited a lower sensitivity: 15% (6%–28%). The N‐ and Z‐tests both differed significantly when compared to the J‐test (*p* < .001), but they did not differ when compared with each other (*p* = .8). The specificities were found to be 90% (78%–97%) for the J‐test, 98% (89%–100)% for the N‐test and 90% (78%–97%) for the Z‐test. The tests did not differ significantly in terms of specificity (*p* = .2). The sensitivity and specificity for each test can be seen in Figure 1B and Table 1.

Upon comparing the effect on the positive predictive value (PPV) when the prevalence of positive patients ranged between 0% and 25%, there was no noteworthy difference found between the tests. The graphical illustration of this is presented in Figure 1C.

## 4 DISCUSSION

In this study, we have evaluated and compared three POC antibody tests designed to detect specific IgG and IgM antibodies against SARS‐CoV‐2. Due to the high contagiousness of SARS‐CoV‐2,^8^ being able to identify patients who have undergone COVID‐19 infection can be valuable during the process of differential diagnosis and for studying routes of transmission. A plethora of antibody tests have been developed for this purpose, but their sensitivity and specificity need to be validated in order for them to be used in routine clinical practice. Of the three evaluated tests, we found no significant differences between the tests regarding the sensitivity to detect IgG antibodies. Furthermore, there was no noteworthy difference between the tests when looking at the effect of prevalence on the PPV.

A similar design has been employed in other studies, and while the sensitivity for detecting antibodies in convalescent samples varies between brands, the sensitivity for detecting antibodies in samples taken in the acute phase of the infection is generally low.^4^, ^6^ As such, RDT POC antibody tests might not be suitable for confirming active infection in the emergency care setting. However, a positive IgG result from a test with high specificity can still potentially be used to *rule out* active infection. When compared to the results on the sensitivity to detect IgG against SARS‐CoV‐2 in convalescent samples from previous studies,^4^, ^5^, ^6^ all three of the evaluated tests in this study had a relatively high sensitivity. A potential reason for this could be that we have evaluated the tests using convalescent serum samples taken at least 28 days post RT‐PCR verified diagnosis. This might indicate that the formation of antibodies can take longer than the previously reported 2–3 weeks.

This study was conducted with a design resulting in a study percentage of samples positive for COVID‐19 of 48%. This does not however mimic the reality for this disease as the estimated prevalence of COVID‐19 in most populations is considerably lower. The control patients in this study were chosen due to their medical history of a similar respiratory infection as patients with COVID‐19. Sera from control patients were collected 4–6 weeks after onset of symptom, which is the same time frame used for the serum samples obtained from the patients with COVID‐19.

As for our results, there were some discrepancies between the tests that need to be discussed. There were three samples that evoked a positive IgG response in the N‐test but that were negative in the J‐ and Z‐test. Similarly, there was one sample that was negative in the J‐test but that was positive in the two other tests and there were two samples that were negative when tested with the Z‐test but that were positive when tested with the two other tests. For the specificities, each test detected IgG in one pre‐COVID‐19 sample but interestingly these false positives were discordant across the three tests.

Using RT‐PCR as a reference method for determining the sensitivity and specificity to detect antibodies is not without problems. This approach assumes that all RT‐PCR positive patients develops antibodies, which is not necessarily true. A potential explanation as to why some RT‐PCR positive patients tested negative for IgG antibodies might be the novel finding of T cell‐mediated immunity against SARS‐CoV‐2.^9^ If immunity can be acquired through the means of T‐cells, it is possible that the patients testing negative for IgG‐antibodies are truly seronegative. There were however still discrepancies between the three tests which can also be explained by low antibody titers. An alternative to the use of sensitivity and specificity is instead reporting the results in terms of *positive percent agreement* (PPA) and *negative percent agreement* (NPA). These are calculated in the same way that sensitivity and specificity are, but more properly illustrate that we are comparing the agreement between two diagnostic methods of uncertain true sensitivity and specificity.^10^ However, as RT‐PCR has become an established gold standard for many authors, for the sake of consistency with the reported findings from similar studies,^4^, ^5^, ^6^ we chose to also use sensitivity and specificity for the observed results in this study.

Another factor potentially affecting seroconversion is disease severity. It has been shown that a more severe course of COVID‐19 correlates to higher levels of antibody production.^11^ Of the 47 included RT‐PCR positive patients, 23 were admitted. About 77% (*n* = 36) of the included patients with COVID‐19 in this study had a mild disease course. Of the six discordant samples mentioned above, five came from patients with mild symptoms. The observed differences in sensitivity was thus likely due to relatively low antibody levels in some individuals with a mild course of COVID‐19.

Since the sensitivity for detecting IgG ranged between 85% and 96% between the three tests, we wanted to examine how this would impact the PPV. As seen in Figure 1C, there was no relevant difference in PPV even when plotting against a low prevalence. As the hypothesized prevalence of COVID‐19 increases, the difference in PPV decreases. At a realistic prevalence of, for example, 10%, all three of the evaluated tests have a similar, and for clinical practices usable PPV of 83%–84%.

In addition to IgG this study also evaluates the ability to detect IgM for the three tests. All serum samples were collected a minimum of 28 days after diagnosis via RT‐PCR testing. However, we cannot rule out the possibility that patients may have been infected before RT‐PCR diagnosis. As the initial IgM response decreases over time, this could explain the relatively low sensitivity for IgM found for the three tests in this study. To accurately measure the IgM response the study should have been designed differently to include patients closer to the RT‐PCR verified diagnosis. This, however, would have hampered our main objective which was to evaluate the presence of antibodies in convalescent samples. Some of the tested RT‐PCR positive serum samples used to evaluate the three tests have also been used for the same purpose to evaluate the Z‐test in another study. The sensitivity for detecting IgM in samples obtained from patients within two weeks of symptom debut was in that study 63%.^12^

A limitation of the present work is the relatively small cohort of study subjects included both in the RT‐PCR positive group and the negative control group. This needs to be taken into consideration when evaluating both sensitivity and specificity. Furthermore, the tests were only evaluated using convalescent sera collected 4–6 weeks after RT‐PCR verification of COVID‐19. We can thus not be certain that the same sensitivities would be observed if the tests were evaluated using convalescent sera collected at a later time point. It would have been of interest to perform the same evaluation using sera from the same patients collected at 3, 6, and 12 months after RT‐PCR diagnosis. On the other hand, we consider the selection of the control patients a strength of this study as they have experienced similar respiratory infections as the patients in the SARS‐CoV‐2 RT‐PCR positive group. Lastly, we did not also evaluate the tests using whole blood. It would have been interesting to investigate whether or not the observed sensitivities for the tests would have been the same if provoked with whole blood rather than sera. In other studies evaluating different RDT POC antibody tests, the observed sensitivities for detecting SARS‐CoV‐2 specific IgG in whole blood were similar to those observed in plasma.^13^, ^14^

## 5 CONCLUSIONS

In conclusion, this study showed that all three tests demonstrated similar performances in detecting SARS‐CoV‐2 specific IgG and that all of them have sufficient sensitivity and specificity to be used in routine clinical practice.

## CONFLICT OF INTERESTS

All the authors declare that there are no conflict of interests.

## AUTHOR CONTRIBUTIONS

*Conceptualization*: Magnus Rasmussen, Adam Linder, and Torgny Sunnerhagen. *Data acquisition*: Rasmus Strand, Louise Thelaus, and Nils Fernström. *Data analysis*: Louise Thelaus, Rasmus Strand, Nils Fernström, and Ylva Lindroth. *Writing*: Rasmus Strand, Louise Thelaus, and Nils Fernström. *Editing*: Rasmus Strand, Louise Thelaus, Magnus Rasmussen, and Torgny Sunnerhagen. *Supervision*: Magnus Rasmussen and Adam Linder.

### PEER REVIEW

The peer review history for this article is available at https://publons.com/publon/10.1002/jmv.26913

## Data Availability

The gathered data supporting the findings of this study are available from the authors upon request.
